# Effects of Processing Temperature on Color Properties of Dry-Cured Hams Made without Nitrite

**DOI:** 10.3390/foods5020033

**Published:** 2016-04-29

**Authors:** Giovanni Parolari, Agnese Aguzzoni, Tania Toscani

**Affiliations:** 1 Experimental Station for the Food Preserving Industry, Viale F. Tanara 31A, 43121 Parma, Italy; agnese.aguzzoni@gmail.com; 2 Consortium for Parma Ham, Largo Calamandrei, 1A 43121 Parma, Italy; tania.toscani@prosciuttodiparma.com

**Keywords:** meat, color, food additives, processing

## Abstract

Dry cured hams were investigated for their ability to develop red color even at low temperature (3–4 °C) and in the absence of added nitrites; results were compared with those obtained from nitrite-free hams made at conventional warm maturing temperatures. Colorimetric parameters (L*, a*, b*, and hue) and concentration of the main pigments Zn protoporphyrin IX (ZnPP) and heme were measured at three stages of preparation (six, nine, and 12 months), showing that red color was successfully formed at low temperatures, though at a slower rate and less intensively than under warm conditions. Major differences in the pattern of color development were found with the two processing temperatures. While the typical features of an enzyme-dependent mechanism, with a progressive drop in enzyme activity paralleling the synthesis of Zn protoporphyrin IX, were observed at warm temperatures, the same did not occur in cold-made hams, where the enzyme activity was almost unchanged throughout the process. These results, along with data from a descriptive sensory analysis, are supportive of a non-enzymatic mechanism leading to ZnPP (hence the red color) under cold conditions, with an estimated three-month delay compared with nitrite-free hams manufactured in a warm maturing regimen.

## 1. Introduction

Nitrite as a food additive has been in the spotlight for decades because of its broad implications in meat technology, including its conflicting effects in the context of consumer health: it acts as a beneficial microbial preservative [1] but also a source of potentially harmful *N*-nitroso compounds [2]. Reviewing nitrite’s functions, Honikel [3] pointed out that with no other molecule exerting the same functions, discontinuing its addition in meats would require the combined use of several chemical and technological strategies. In this respect, dried hams in the last few years have become a case study for their ability to develop red color even without nitrites, as is the case with Parma hams, traditionally prepared with sea salt only, excluding any other additives. After Zn protoporphyrin IX was identified as the main contributor to color in nitrite-free hams [4], studies have addressed the reaction by which, in an inherently endogenous mechanism, the muscle heme undergoes a progressive substitution of zinc for iron. Using pork meat as a model, Wakamatsu *et al.* [5] documented the ability of the muscle ferrochelatase to catalyze the iron displacement from the heme moiety, a key step prior to the eventual insertion of zinc, leading to Zn protoporphyrin IX. The enzymatic pattern of the ZnPP synthesis was further investigated by Parolari *et al.* [6], who assayed the enzyme in several thigh muscles, finding different activities in red (high activity) *vs.* white (low activity) thigh muscles. The enzyme was shown to behave differently when the legs were submitted to dry-salting, explaining the heterogeneous ZnPP contents found in finished hams [6,7].

While these and other [8] investigations have pointed to ferrochelatase as a crucial reactant in the ZnPP development pathway, a non-enzymatic mechanism has been postulated as an independent, additional route to the synthesis of the pigment in nitrite-free hams. Using pork slurries and slices of Parma ham as substrates, Becker *et al.* [9] demonstrated that ZnPP could be formed even in the presence of a chelatase inhibitor, such as *N*-methyl-mesoporphyrin. Observing that the enzyme activity faded by the end of maturation, they suggested that ZnPP could result from a concurrent enzymatic and non-enzymatic pattern, with the former prevailing in the early maturing phase and the latter in the subsequent aging phase. The removed iron was proven [10] to end up in an irreversible colloidal form of Fe (III) hydroxide, making it easy for the free zinc ion to fit into the loose heme pouch. This reaction would be encouraged by partial hydrolysis of the globin moiety, supported by the proteolytic muscle enzymes [11].

If the non-enzymatic pathway could be demonstrated to occur even at chilling temperature, or at values below the threshold temperature required for ferrochelatase activation [12], then a new approach would be available for manufacturing of nitrite-free dried meats, combining the advantages stemming from a low-temperature process with the achievement of a stable red color through ZnPP. For example, a low-temperature regimen would enable improved microbial control in such cases where low-salt meats are prepared, in compliance with current nutritional recommendations [13].

Therefore, in the present study the development of ZnPP in nitrite-free hams was investigated at a chilling (3–4 °C) temperature, maintained until the end of manufacturing. The resulting color properties and the potential use of the low-temperature option in the meat industry are described.

## 2. Experimental Section

### 2.1. Samples

Hams (*n* = 20) belonging to the same production batch were obtained from a local manufacturer after completion of the cold-processing phase for dry cured ham, or at three months after the manufacturing process was started. They had previously been dry-salted in compliance with current guidelines for production of Parma ham [14], *i.e*., only with marine salt and no nitrite or nitrate. Two hams were immediately analyzed, while the remaining pieces were randomly assigned to two groups of nine hams each. One group was further processed in agreement with the specifications for Parma ham for a total of nine months, comprising a maturing stage at T = 13–15 °C for three months and an aging phase of six months at T = 15–18 °C until the completion of the process, at 12 months following the salting date. The other group of hams was kept chilled at T = 3–4 °C and RH = 65%–75% throughout the same nine-month period. After the first maturing period, corresponding to six months after salting, the hams were covered with a spreadable mince made of pork fat and salt to prevent excessive drying of lean meat. Samples for the analyses were removed from each of three hams per group at six, nine, and 12 months of age and consisted of a 100-gram core portion of the *biceps femoris* (BF) and *semimembranosus* (SM) muscles, obtained by cutting the hams perpendicular to the femur bone, at the joint level.

### 2.2. Reagents and Chemicals

Hemin (heme), protoporphyrin IX (PPIX), Zn protoporphyrin IX (ZnPP), ethyl acetate, triton X-100, trizma^®^ base, ATP dipotassium salt, zinc sulfate, EDTA, Tween^®^ 80, and potassium ferricyanide were purchased from Sigma-Aldrich (St. Louis, MO, USA). Ammonium acetate, nitric acid 65%, glycerol, potassium chloride, and sodium chloride were from Carlo Erba (Milan, Italy). HPLC grade methanol, acetic acid glacial, and trichloroacetic acid were from VWR BDH Prolabo (Lutterworth, UK). Reagents and chemicals were stored according to the suppliers’ instructions.

### 2.3. Instrumental Color Measurements

The CIE L*a*b* parameters were recorded on the muscle surface just after the sample removal by a portable Konica Minolta CM-700d reflectance spectrocolorimeter (Minolta, Osaka, Japan). Readings were performed using diffuse illumination, with D65 as illuminant and 10° as standard observer, 8° geometry, and 8 mm port size, specular component excluded. For each muscle, the measurements were recorded as the average of 10 independent readings of the exposed muscle area.

### 2.4. Chemical Analyses

Three major proximate muscle components (moisture, salt, and total nitrogen) were obtained as previously described [6]. 

The proteolysis ratio (PR), a marker of protein breakdown resulting from enzyme activity, was determined as the percent ratio between non-protein nitrogen (NPN) left after treatment of the aqueous extract with trichloroacetic acid [15] and the total nitrogen (TN) in the meat. Accordingly, 20 g minced muscle was mixed with 180 mL water and homogenized for 1 min, then centrifuged at 10,000 rpm for 15 min at 5 °C. After filtration, 50 mL of the solution were added to 50 mL of 10% trichloroacetic acid (TCA) in water and allowed to react for 30 min, then centrifuged as described above. The filtered solution (40 mL) was eventually analyzed for total nitrogen and the proteolysis ratio calculated as PR = (NPN/TN) × 100.

The muscle pH was obtained by inserting a glass electrode attached to a pH-meter (pH300, Hanna Instruments, Houston, TX, USA) into the minced meat at room temperature.

Water activity (a_w_) was measured by LabMaster (Novasina, Lachen, Switzerland), according to the International Standard ISO 21807: 2004 [16].

### 2.5. Pigment Extraction and Analysis

Extraction of red pigments i.e. heme, Zinc protoporphyrin IX (ZnPP) and protoporphyrin IX (PPIX) was carried out in agreement with the procedure given by Wakamatsu *et al.* [17], with the following minor changes. The finely ground ham (1 g) was added to 20 mL of an ethyl acetate:acetic acid:water 72:18:10 solution (v/v) and the mixture was homogenized at 9500 rpm for 2 min then extracted for 30 min in the dark, at 1–4 °C. After centrifuging at 6000 rpm for 10 min at 4 °C, the supernatant was collected in a volumetric flask, filtered through PTFE 0.45 μm syringe filter (Agilent Technologies, Basel, Switzerland) and diluted with a methanol:ammonium acetate solution (84:16, v/v, pH 5.16). The pigments were separated by injecting the extract (50 μL) into an HPLC equipment (Agilent 1100 series, provided with an X-Terra C18 column), with the eluent (methanol:ammonium acetate 84:16, v/v, pH 5.16) flowing isocratically at 1.0 mL/min. Pigments were quantified by their absorption at 400 nm (UV/visible detector, Agilent 1200) and, for ZnPP and PPIX, also by fluorescence (Agilent 1100) at excitation/emission wavelengths of 420/590 nm and 400/630 nm, respectively. The analysis was performed in both muscles (BF and SM) collected in the core samples and results were expressed as the average of two replicates.

### 2.6. Zn-Chelatase Activity

The enzyme activity was determined according to the method described by Benedini *et al.* [12], modified as follows. The ground sample (8 g) was added with 50 mL of extraction buffer (pH 8) at 4 °C and homogenized at 5000 rpm for 2 min. The extraction buffer consisted of a water solution of tris-HCl 20 mmol/L, glycerol 20% (w/v), Triton X-100 1% (w/v), and KCl 0.8% (w/v). After stirring (30 min, 2 °C) and centrifuging at 15,000 rpm for 10 min at 4 °C, the supernatant was collected and stored at 4 °C in the dark, and the pellet was re-extracted with 30 mL of the same buffer. The supernatants were finally joined, filtered through Whatman paper n° 40 (Whatman Intl. Ltd, Maidstone, UK), and submitted to enzyme assay. For this purpose, the meat extract (500 μL) was added to a reaction tube with 250 μL of ZnSO_4_ 400 μmol/L, 50 μL of protoporphyrin IX 1 mol/L, and 200 μL of adenosine triphosphate dipotassium salt (ATP) 25 mmol/L in NaCl 200 g/L. After incubation at 37 °C for 45 min in the dark, 35 μL of EDTA 50 mmol/L were added to stop the reaction and the tubes were cooled on ice. An equal volume of 96% (v/v) ethanol was then added and the resulting solution centrifuged at 13,000 rpm for 10 min. The clear supernatant was submitted to fluorescence analysis by scanning a fluorescence spectrometer (LS30, Perkin-Elmer, Waltham, MA, USA) from 500 to 700 nm with excitation at 415 nm and emission at 590 nm. Each meat extract was assayed twice against a blank consisting of the reaction mixture with 35 μL of EDTA 50 mmol/L added. A calibration curve for ZnPP was obtained in the 0.125–5 μmol/L range by diluting a concentrated solution of ZnPP (2 mol/L in THF) in a buffer solution made up of the extraction buffer (250 mL/L), 360 mmol/L tris-HCl buffer (150 mL/L), distilled water (100 mL/L), and 96% v/v ethanol (500 mL/L). The resulting regression equation (*R*^2^ = 0.998) was used to calculate the enzyme activity in the extract. Accordingly, one unit (U) of Zn-chelatase activity was defined by the enzyme amount catalyzing the conversion of 1 nmol of PPIX in 1 min. The assay was performed in both muscles (BF and SM).

### 2.7. Sensory Analysis

Hams were evaluated by quantitative descriptive analysis according to procedures described by Meilgaard *et al.* [18]. The panel, comprising eight assessors with previous experience in visual evaluation of dried meats, were trained [19] in three preliminary sessions for the use of the sensory card, comprising three visual attributes, *i.e*., red, purple, and brown color intensities. To rate the samples, the panel members were instructed to use a non-structured (0–9) scoring scale, where 0 meant the absence of and 9 the greatest possible intensity of the attribute, respectively. To aid the panel with the scoring scale, the attributes were anchored to their respective upper extremes (score 9) by dried hams selected to represent the pertinent attributes (Table 1). The samples were evaluated within 2 min of storage of the cut slice, displayed at ambient temperature.

### 2.8. Statistical Analysis

The General Linear Model (GLM) procedure was used to estimate the effects of factors (time and temperature of maturation) and interaction terms. Those variables showing significant main effects were further inspected by the *post hoc* Least Significant Difference (LSD) multiple comparison test. All statistics were obtained by the IBM SPSS Statistics v. 22 (Armonk, NY, USA; 2013) package.

## 3. Results and Discussion

### 3.1. Chemical and Physical Properties of Dried Hams

Estimated means from the GLM analysis of proximate composition data are reported in Table 2 for both muscles tested at four sampling times (three, six, nine, and 12 months). Moisture contents decline because of aging, with significant (*p* < 0.01) dehydration occurring in the internal BF, but not in the outer SM muscle, whose moisture reaches a steady 57% after just three months of processing. Temperature affects moisture in both muscles, with lower (*p* < 0.01) water content in hams matured at 16 °C than in their cold-rested counterparts.

As a consequence of time-related dehydration, salt increases after the third month in both muscles, reaching its maximum at 9–12 months. Salt is affected by temperature in the SM muscle, where greater concentrations at 16 °C, combined with a steady increase throughout the manufacturing time, result in a significant (*p* < 0.05) time × temperature interaction. Salt exhibits stable values in the outer SM fraction, whereas in the BF muscle it is significantly lower in the first than at subsequent sampling times, suggesting that three months of processing are insufficient for sodium chloride to be fully equalized inside the leg. Changes in moisture and salt content are clearly reflected in regularly decreasing water activity (a_w_) values, with the same end point (a_w_ = 0.924) obtained in both muscles at 12 months of processing. On average, a_w_ measurements taken in the hams at 16 °C are significantly lower (*p* < 0.01) than in their cold-matured peers, in accordance with the greater dehydration effected by warm maturation [20].

The proteolysis ratio (PR), a marker of non-protein nitrogen yielded by enzymatic cleavage, is shown to be time-dependent in the BF muscle, reaching a final level of 26.4%, or 4.7% more than in the SM muscle, where values remained unchanged after the first sampling time. As can be observed in the temperature-related data, a temperature of 16 °C resulted in greater PR in both muscles, with the BF largely exceeding the outer SM portion. Values were in agreement with those reported in previous studies, where proteolysis patterns were proven to be temperature- and muscle-dependent [21]. The trends found in the PR of the BF muscles are paralleled by a slight increase (*p* < 0.05) in their pH values, and a rise is likewise observed in the average pH of the same muscle when measured at higher *vs.* lower temperature. A relationship of pH to non-protein nitrogen has been reported in long-matured dried hams, and the ability of a few molecules released by protein breakdown (free amino acids, ammonia) to raise the meat pH has been postulated [22]. In the present study, greater pH values found in the inner BF muscle could have been triggered by protein cleavage, increased by aging time and temperature.

The data in Table 3 enable a more focused evaluation of the temperature effect in the BF muscle of finished hams. They show differences in moisture, a_w_, and PR as result of the processing temperature, with significantly (*p* < 0.01) lower a_w_ and higher proteolysis in the hams at 16 °C. While such differences are not surprising, it is noteworthy that PR could reach such a large end point as 24.9% even under permanently cold conditions. If confirmed, this finding would entail that other mechanisms than those ascribed to muscle enzyme activity can contribute to protein disruption, since the proteolytic activity of aging hams is strictly time- and temperature-dependent [21].

### 3.2. Colorimetric Measurements

Average tristimulus color measurements are reported in Table 4 as a function of maturing time and temperature. They show non-significant changes in brightness (L*) in the BF muscle, whereas in the SM portion L* declines after an uptrend at 6–9 months, reflecting the uneven moisture changes observed between 3–12 months (Table 2). A significant (*p* < 0.01) temperature effect is shown in the SM and, similarly, in the BF muscle, with lower brightness observed at 16 °C, as a result of greater residual moisture, and, consequently, of lightness [23] in cold- *vs.* warm-processed samples. When a* (red) and b* (yellow) are considered, a time-related U-shaped change is observed for both parameters, with a rebound occurring at 12 months. A significant (*p* < 0.05) temperature effect is shown for a* in both muscles, with the upper processing temperature (16 °C) associated with redder color intensity. The role of temperature is further evidenced in the hue variable (hue angle), a combination of a* and b*, inversely related to redness. Hue values in both muscles are significantly lower at 16 °C, confirming that raising the temperature yields a more intensively red muscle, compared with that achieved by cold processing.

**Figure 1:**
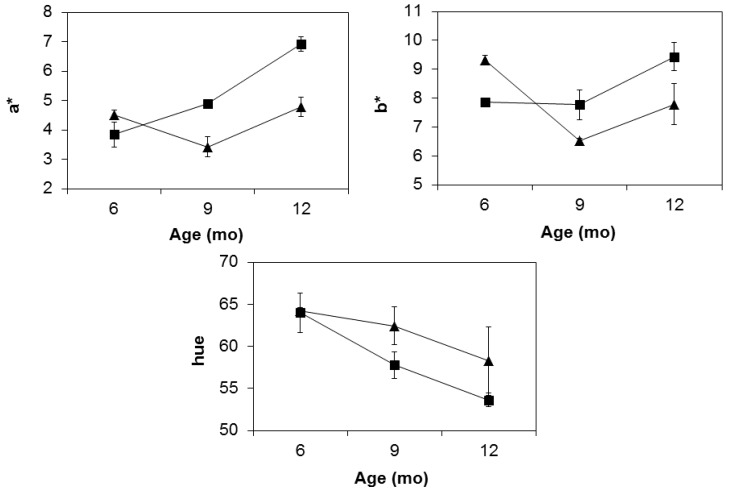
Changes of colorimetric a*, b*, and hue parameters in the BF muscle at three maturing times as a function of temperature: (▲) T = 4 °C, (■) T = 16 °C.

The combined effects of temperature and aging time (6–12 months) on a*, b*, and hue are graphically described in Figure 1 for the BF muscle, a key ham fraction, where color formation has been reported to take longer to achieve and is overall less stable compared to the outer leg muscles [6]. Measurements from nine to 12 months exhibit an overall increase for a* (*p* < 0.05) and, to a lesser extent, b*, regardless of temperature. As a result, hue in the end product drops to <54 in warm-made hams, matching the trend typically observed in ready-to-eat, nitrite-free hams [24]. Over the same time span, their cold-processed counterparts exhibit a progressive hue decrease to 58, the same value found in warm-kept hams at nine months, or three months earlier. The delayed pattern of cold hams toward redness is explained by the different behavior of a* and b* as influenced by maturing temperatures. As shown by their significant (*p* < 0.05) interaction (Table 4), the two colorimetric parameters exhibit diverging trends between six and nine months according to temperature, with stable (b*) or rising (a*) values at 16 °C, as opposed to a sharp fall in their cold-stored counterparts. Consequently, the hue in warm hams already started to fall at six months, while it remained almost flat in their cold peers. However, the interactive trend became additive after the ninth month, when a* and b* increased at both temperatures, maintaining a stable gap in values until the end of manufacturing, corresponding to approximately three months of aging. The data suggest that, for cold-processed hams to achieve the same red values as in warmed hams, an extra storage time of at least three months should be provided.

### 3.3. Analysis of Pigments

**Figure 2:**
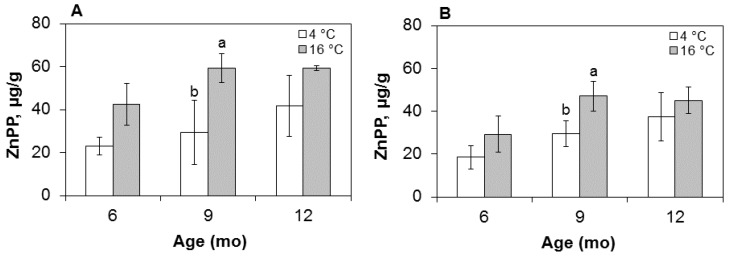
ZnPP concentration in the (**A**) BF and (**B**) SM ham muscles at three sampling times (six, nine, and 12 months) and two maturing temperatures (4 °C and 16 °C). Data in µg/g of dry matter. For each pair of bars, different letters denote a significant (*p* < 0.05) temperature effect.

Red color in nitrite-free dried hams has been demonstrated to be dependent on two natural pigments such as ZnPP and heme [4,25], with PPIX playing a minor role. Accordingly, ZnPP and heme in this work were quantified as the two major color determinants and their concentrations evaluated as a function of the two independent factors under investigation, *i.e*., maturing time and temperature. Results, reported in Table 5 for the three natural pigments and their sum, indicate an increase (*p* < 0.05) over time in both muscles for ZnPP, with heme and PPIX showing non-significant changes. As a net result, the sum of pigments increased over time, reaching a maximum in the end product at 12 months. A temperature effect was observed for ZnPP, which increased significantly (*p* < 0.01) in both muscles. As shown in Figure 2, where the pigment contents are graphically reported according to temperature and maturation time, ZnPP in the BF muscle is greater at the upper processing temperature, with a statistically significant difference (*p* < 0.05) at nine months, and a final concentration of 59 µg/g at 16 °C, compared with 40 µg/g in the cold-stored muscle. ZnPP is likewise increased in the outer SM portion, which exhibits a similar time- and temperature-dependent pattern but at generally lower concentrations. Interestingly, ZnPP in the cold-stored BF muscle exceeded its cold-matured SM counterpart at 12 months, reaching an end value of 40 µg/g, *i.e*., the same as in the SM at 16 °C.

In both muscles, the ZnPP growth resulted in an increased total of pigments at 12 months (Table 5) and fits the trend of hue (Figure 1) at both temperatures, progressively falling to lower values and hence producing a redder color. Based on the pigment data, it may be concluded that color in nitrite-free hams is contributed, regardless of the processing temperature, by two natural pigments such as ZnPP and heme.

### 3.4. Enzyme Activity

**Figure 3:**
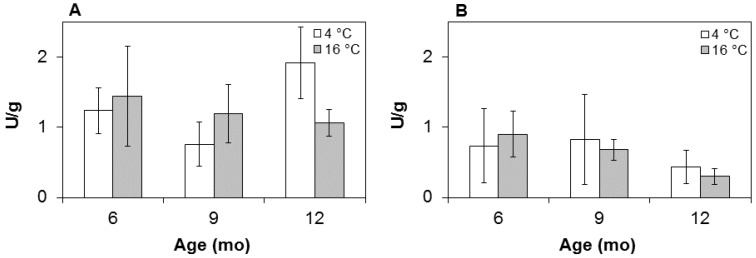
Enzyme activity in the (**A**) BF and (**B**) SM ham muscles at three sampling times (six, nine, and 12 months) and two maturing temperatures (4 °C and 16 °C). Data (U/g) in (nmol·min^−1^)/g dry matter.

Results of the enzyme assays, performed in both muscles, are reported in Figure 3 as related to maturing time and temperature. They show a decreasing activity over time in hams held at 16 °, but not in cold-stored hams, where the enzyme does not follow an obvious trend line. In agreement with previous measurements of porcine muscles [6], the average chelatase activity as assayed in these hams was greater (*p* < 0.05) in the internal BF (1.3 U/g dry matter) compared with the external SM (0.6 U/g dry matter) muscle. This may reflect natural differences in the biochemical pattern of thigh components, where the *biceps femoris* behaves intermediately between a typical white muscle such as the s*emimembranous* and a red one, like the dark *semitendinosus* [26]. Matched with the ZnPP development pattern (Figure 2), the enzymatic activities at 16 °C exhibit an opposite, specular trend over time, with less enzyme left in the hams at 12 months of age. This was especially the case with the SM samples, whose activity is virtually lost at the end of the process, regardless of temperature. In contrast, the enzyme activity in the BF was significantly greater, with no decreasing trend in the cold samples compared with the warm ones. Results are supportive of a non-enzymatic synthesis of ZnPP in cold-processed hams, where the low temperature would exert a protective effect on the enzyme, especially in the inner muscles. Keeping active even later in maturation, the muscle Zn-chelatase would warrant an additional heme conversion to ZnPP at any time in manufacturing, if and when the temperature is raised to levels priming the enzyme activity.

### 3.5. Sensory Analysis

**Figure 4:**
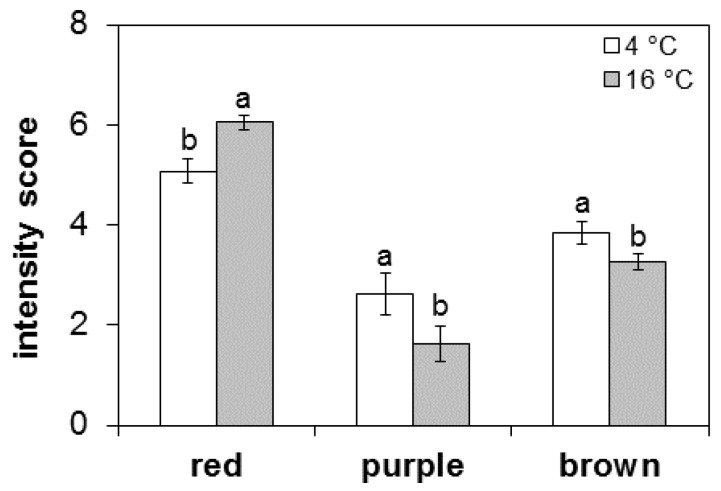
Comparison of intensity scores for hams processed at two maturing temperatures (4 °C and 16 °C). For each couple of bars, different letters denote a significant (*p* < 0.05) temperature effect.

When testing the hams for visual color, the panelists were asked to focus on three attributes chosen to describe (1) the red intensity typically associated with fully matured hams, (2) the purple color delivered by incompletely dried muscle, and (3) the brownish nuance of discolored meat, with the first and third character eliciting acceptance and rejection of the item, respectively. Values, reported in Figure 4 as average panel scores at two temperatures, show significantly (*p* < 0.05) greater red as opposed to lower purple and brown intensities in the hams processed at a higher temperature. These results are in agreement with and add evidence to the beneficial temperature effects on color, where red intensity at 16 °C exceeded the less desirable purple and brown scores. However, though less marked, the red scores assigned to the cold-stored hams were considerably larger than their purple and brown ratings, meaning that red pigments formed at 12 months offset the unstable purple and brown nuances typically associated with pork myoglobin and the oxidized metmyoglobin, respectively, of uncured or nitrite-free meats.

## 4. Conclusions

With no alternative additives allowed for nitrite replacement, obtaining red color without nitrosating agents is a challenging task for meat processors. In this study, nitrite-free dried hams developed a stable color through the endogenous synthesis of red Zn-protoporphyrin IX, according to a natural, enzyme-dependent process conducted at a warm maturation temperature. Unexpectedly, the same pigment was formed even by holding the hams at chilling temperature throughout manufacturing, which would support a non-enzymatic synthesis of the pigment in nitrite-free hams, although a residual enzyme activity occurring even at a low temperature could not be excluded. Though still unexplored, and slower to accomplish than its warm counterpart, the cold-temperature process would open new perspectives in dry-cured meat production, supporting those producers interested in a no-additive, low-salt alternative. These results show that extending the cold stage until the water activity drops to safe levels does not prevent the red pigment Zn protoporphyrin IX from forming, even if it occurs later than in conventionally cured meat. Furthermore, the heme-converting enzyme, which remains active during chilling, yields extra pigment, hence color, if and when the temperature is raised later in aging. More studies are needed to evaluate the cold-forming color process as related to intrinsic pork muscles, and shifts in physical (pH, a_w_) and technological (relative humidity, salt diffusion rate) properties typically occurring in industrial practice.

## Figures and Tables

**Table 1:** Sensory evaluation of dried hams. Reference standards were used to anchor the upper scale extremes for three visual attributes.

Visual Attribute	Description	Reference Standard	Score (0–9)
Red	The attractive color of long-matured dried hams	Dried ham after 24 months maturation; heme <20 µg/g and ZnPP > 60 µg/g	9
Purple	The color of half-processed hams	Ham at the end of the resting phase; heme > 80 μg/g and ZnPP < 10 µg/g	9
Brown^1^	The unattractive color of dried hams after air exposure	Dried ham whose pigments have turned into metmyoglobin ^1^	9

^1^: This sample was prepared by immersing a thick slice of 24-month dry-cured ham in 1% potassium ferricyanide for 1 min then packaging it in an oxygen-permeable film containing 1% O_2_ and leaving it for 48 h at refrigerator temperature. According to this procedure, made in agreement with AMSA (American Meat Science Association) Guidelines for Meat Color Evaluation (1991), the meat pigments were thoroughly converted into oxidized brown metmyoglobin.

**Table 2:** GLM analysis of chemical components (dependent variables) of hams as related to processing age and temperature (fixed factors). Estimated marginal means ± standard error, significance of factors, and their interaction (A × T). Analyses carried out in two ham muscles: *biceps femoris* (BF) and *semimembranosus* (SM).

Component	Muscle	Age (months)				*P*_A_	Temperature (°C)		*P*_T_	*P*_A × T_
		3	6	9	12		4	16		
Moisture ^1^	BF	67.3 ± 0.7 a	66.5 ± 0.4 a	65.2 ± 0.4 b	63.9 ± 0.4 c	**	65.9 ± 0.3 a	64.5 ± 0.3 b	**	*
	SM	57.3 ± 1.9	55.6 ± 1.1	57.1 ± 1.1	57.5 ± 1.1	NS	58.1 ± 0.9 a	55.4 ± 0.9 b	*	NS
Salt ^1^	BF	4.6 ± 0.2 d	6.1 ± 0.1 b	6.4 ± 0.1 a	5.7 ± 0.1 c	**	6.1 ± 0.1	6.1 ± 0.1	NS	*
	SM	4.3 ± 0.2 c	5.1 ± 0.1 b	5.3 ± 0.1 ab	5.5 ± 0.1 a	*	5.0 ± 0.1 b	5.5 ± 0.1 a	**	*
S/M ^2^	BF	6.8 ± 0.2 c	9.2 ± 0.1 b	9.8 ± 0.1 a	9.0 ± 0.1 b	**	9.2 ± 0.1	9.4 ± 0.1	NS	**
	SM	7.6 ± 0.4	9.2 ± 0.2	9.3 ± 0.2	9.5 ± 0.2	NS	8.7 ± 0.2 b	10.0 ± 0.2 a	**	*
Protein ^1^	BF	23.1 ± 0.5	27.2 ± 0.3	26.9 ± 0.3	26.2 ± 0.3	NS	26.7 ± 0.2	26.8 ± 0.2	NS	NS
	SM	28.6 ± 0.7	29.4 ± 0.4	30.8 ± 0.4	30.5 ± 0.4	NS	30.2 ± 0.3	30.2 ± 0.3	NS	NS
a_w_	BF	0.952 ± 0.003 a	0.938 ± 0.002 b	0.931 ± 0.002 c	0.924 ± 0.002 d	**	0.938 ± 0.001 a	0.924 ± 0.001 b	**	NS
	SM	0.939 ± 0.002 a	0.931 ± 0.001 b	0.928 ± 0.001 bc	0.924 ± 0.001 c	*	0.935 ± 0.001 a	0.920 ± 0.001 b	**	NS
Proteolysis Ratio (PR)	BF	20.7 ± 1.4 b	23.5 ± 0.8 b	23.5 ± 0.8 b	26.4 ± 0.8 a	*	22.4 ± 0.7 b	26.6 ± 0.7 a	**	NS
	SM	21.2 ± 0.7	21.6 ± 0.4	21.9 ± 0.4	21.7 ± 0.4	NS	20.2 ± 0.4 b	23.3 ± 0.4 a	**	NS
pH	BF	5.75 ± 0.04 b	5.79 ± 0.02 b	5.86 ± 0.02 a	5.86 ± 0.02 a	*	5.81 ± 0.02 b	5.87 ± 0.02 a	*	NS
	SM	5.76 ± 0.06	5.76 ± 0.04	5.80 ± 0.04	5.79 ± 0.04	NS	5.78 ± 0.03	5.79 ± 0.03	NS	NS

*P*_A_: significance of the age effect; *P*_T_: significance of the temperature effect; *P*_A×T_: significance of the age × temperature interaction. Asterisks denote significance * = *p* < 0.05, ** = *p* < 0.01. NS = not significant (*p* > 0.05); a, b, c, d = within each experimental factor, different letters along rows denote significance (LSD multiple difference test); ^1^: moisture, salt, protein expressed in grams/100 grams wet sample; ^2^: S/M = salt*100/moisture.

**Table 3:** Proximate components of dried hams after 12 months of aging (BF muscle). Average values for temperatures at 4 °C and 16 °C.

Component	Temperature		*P*
	4 °C	16 °C	
Moisture ^1^	65.4 ± 0.4	62.4 ± 0.5	**
Salt ^1^	5.6 ± 0.2	5.9 ± 0.2	NS
a_w_	0.933 ± 0.003	0.915 ± 0.07	*
Proteolysis Ratio	24.9 ± 0.8	28.0 ± 0.7	**

*P*: significance of the temperature effect. Asterisks denote significance * = *p* < 0.05: ** = *p* < 0.01. NS = not significant (*p* > 0.05); ^1^: moisture and salt expressed in grams/100 grams wet sample.

**Table 4:** GLM analysis of dried hams’ color parameters (dependent variables) as related to age and temperature (fixed factors). Estimated marginal means ± standard error, significance of factors, and their interactions (A × T). Measurements made in the *biceps femoris* (BF) and Semimembranosus (SM) muscles.

Color Parameter	Muscle	Age (months)				*P*_A_	Temperature (°C)		*P*_T_	*P*_A × T_
		3	6	9	12		4	16		
L *	BF	51.7 ± 2.0	51.2 ± 1.2	51.0 ± 1.2	49.9 ± 1.2	NS	51.1 ± 1.0	50.3 ± 1.0	NS	NS
	SM	42.7 ± 1.5 b	49.1 ± 0.8 a	47.7 ± 0.8 a	44.8 ± 0.8 b	**	49.3 ± 0.7 a	45.1 ± 0.7 b	**	NS
a *	BF	8.2± 0.3 a	4.2 ± 0.2 c	4.2 ± 0.2 c	5.9 ± 0.2 b	**	4.2 ± 0.1 b	5.2 ± 0.2 a	**	*
	SM	6.3 ± 0.5 ab	5.0 ± 0.3 c	5.2 ± 0.3 bc	6.4 ± 0.3 a	*	5.0 ± 0.2 b	6.1 ± 0.2 a	**	NS
b *	BF	13.1 ± 0.5 a	8.6 ± 0.3 b	7.1 ± 0.3 c	8.6 ± 0.3 b	*	7.9 ± 0.2	8.4 ± 0.3	NS	*
	SM	10.1 ± 0.9	7.9 ± 0.5	7.2 ± 0.5	7.9 ± 0.5	NS	7.9 ± 0.4	7.2 ± 0.4	NS	NS
hue	BF	58.2 ± 2.6 ab	64.1 ± 1.7 a	60.1 ± 1.7 ab	55.9 ± 1.5 b	*	61.6 ± 1.2 a	58.5 ± 1.2 b	*	NS
	SM	58.0 ± 2.7 a	57.2 ± 1.6 a	53.7 ± 1.6 ab	51.0 ± 1.6 b	*	57.5 ± 1.3 a	49.9 ± 1.3 b	**	NS

*P*_A_: significance of the age effect; *P*_T_: significance of the temperature effect; *P*_A×T_: significance of the interaction age x temperature. Asterisks denote significance * = *p* < 0.05, ** = *p* < 0.01. NS = not significant (*p* > 0.05); a, b, c = within each experimental factor, different letters along rows denote significance (LSD multiple difference test).

**Table 5:** GLM analysis of pigment concentrations (dependent variables) as related to maturing time (age) and temperature. Estimated marginal means ± standard error, significance of factors, and their interactions (A × T).

Pigments ^1^	Muscle	Age (Months)				*P*_A_	Temperature (°C)		*P*_T_	*P*_A × T_
		3	6	9	12		4	16		
Heme	BF	51.9 ± 9.1	48.3 ± 5.3	43.6 ± 5.3	51.9 ± 5.3	NS	52.4 ± 4.3	43.5 ± 4.3	NS	NS
	SM	55.2 ± 7.9	39.8 ± 4.5	39.2 ± 4.5	49.7 ± 4.5	NS	47.0 ± 3.7	38.8 ± 3.7	NS	NS
ZnPP	BF	19.5 ± 8.9 c	32.8 ± 5.1 bc	44.3 ± 5.1 ab	50.5 ± 5.1 a	*	31.4 ± 4.2 b	53.7 ± 4.2 a	**	NS
	SM	16.5 ± 5.3 b	23.8 ± 3.0 b	38.3 ± 3.0 a	41.2 ± 3.0 a	*	28.4 ± 2.5 b	40.4 ± 2.5 a	**	NS
PPIX	BF	1.4 ± 2.4	3.5 ± 1.4	4.6 ± 1.4	41.2 ± 1.4	NS	2.5 ± 1.1	5.6 ± 1.1	NS	NS
	SM	1.9 ± 1.7	5.6 ± 1.0	6.2 ± 1.0	4.4 ± 1.0	NS	6.1 ± 0.8	4.7 ± 0.8	NS	NS
Sum of pigments	BF	72.8 ± 8.6 b	84.6 ± 4.9 b	92.6 ± 4.9 ab	106.4 ± 4.9 a	*	86.3 ± 4.0 b	102.7 ± 4.0 a	*	NS
	SM	73.6 ± 7.9 bc	69.3 ± 4.6 c	83.7 ± 4.6 ab	95.4 ± 4.6 a	*	81.6 ± 3.7	84.0 ± 3.7	NS	NS

*P*_A_: significance of the age effect; *P*_T_: significance of the temperature effect. Asterisks denote significance * = *p* < 0.05; ** = *p* < 0.01. NS = not significant (*p* > 0.05); a, b, c = within each experimental factor, different letters along rows denote significance (LSD multiple difference test); ^1^: Concentration of pigments in µg/g dried substance.
